# Effects of a co-created occupational health intervention on stress and psychosocial working conditions within the construction industry: A controlled trial

**DOI:** 10.3389/fpubh.2022.973890

**Published:** 2022-09-23

**Authors:** Emma Cedstrand, Hanna Augustsson, Magnus Alderling, Néstor Sánchez Martinez, Theo Bodin, Anna Nyberg, Gun Johansson

**Affiliations:** ^1^Unit of Occupational Medicine, Institute for Environmental Medicine, Karolinska Institutet, Stockholm, Sweden; ^2^Medical Management Centre, Department of Learning, Informatics, Management and Ethics, Karolinska Institutet, Stockholm, Sweden; ^3^Center of Occupational and Environmental Medicine, Stockholm Region, Stockholm, Sweden; ^4^Department of Medicine, Universitat Internacional de Catalunya (UIC), Barcelona, Spain; ^5^Department of Public Health and Caring Sciences, Uppsala University, Uppsala, Sweden

**Keywords:** implementation fidelity, professionals, first-line managers, role clarity, pattern mixture model, marginal means model, mental health, evaluation study

## Abstract

**Background:**

Work-related stress problems, i.e., burnout, depression, and anxiety, is a rising global health challenge. Poor mental health also appears to be a challenge for the construction industry, even though the occupational health focus has traditionally been on the physical work environment and musculoskeletal disorders. Yet, studies targeting the organisational level (i.e., work environment, policy) to enhance mental health within the construction industry are scant. Therefore, our first objective was to evaluate the effectiveness of a co-created occupational health intervention on stress and psychosocial working conditions within the construction industry in Sweden. The second objective was to evaluate whether the intervention was implemented as intended, i.e., implementation fidelity. The trial is registered in the ISRCTN clinical trial registry (ISRCTN16548039, http://isrctn.com/).

**Methods:**

This is a controlled trial with one intervention and one matched control group. We co-created the program logic with stakeholders from the intervention group. The essence of the chosen intervention components, duties clarification, and structured roundmaking was enhanced planning and role clarification. We assessed adherence to the intervention and dose delivered (i.e., fidelity). We collected data on the outcomes (role clarity, team effectiveness, planning, staffing, quantitative demands, and the psychosocial safety climate) with online questionnaires at baseline, 12, and 24 months. Marginal means models adjusting for missing data patterns were applied to estimate potential differences in outcomes between groups over time.

**Results:**

Fidelity was considered reasonably high. Yet, we found no intervention effects on the primary outcome stress. All outcomes, except role clarity deteriorated during the trial in the intervention and control group. However, the results indicate a positive effect of the intervention components on professionals' role clarity. The pandemic appears to have negatively affected stress and psychosocial working conditions.

**Conclusion:**

The study's results suggest that co-creating occupational health interventions could be one solution for improved implementation fidelity. More studies are needed to evaluate these intervention components. Also, we recommend researchers of future intervention studies consider using missing not at random, sensitivity analysis.

## Introduction

Work-related stress problems constitute a significant challenge for Western countries today. In Sweden, poor mental health is the most common cause of sick leave. Stress-related diagnoses, such as acute stress reaction and burnout, are the causes of sick leave that, in both women and men, have increased the most in recent years, even though women consistently face a higher risk than men (1, 2). The construction industry is male-dominated (>70% men) and includes both trade workers and professionals (3). It was recently noted that among employees on sick leave in the construction industry, for first-line managers, the risk that the cause for sick leave was stress-related was 25% higher compared to all other occupations. Technical engineers seem to face similar risks; however, this trend is not observed for trade workers (2). Yet, suicide rates are higher among construction trade workers than the male national average in Great Britain (4), Australia, and New Zeeland (5). Also, Matilla-Santander et al. (6) observed a similar trend in Sweden; however, not statistically significant. Hence, poor mental health appears to be a challenge for the construction industry, even though trade workers and professionals seem to have different patterns of poor mental health. A potential consequence of stress among construction workers is increased workplace accidents (7–9). Among construction workers in Sweden, reporting daily stress was associated with an increased risk of being in a severe workplace accident compared to reporting stress seldom or never (7).

Traditionally, the occupational health focus in the construction industry has been on the physical work environment and musculoskeletal disorders (10). However, in line with the above-described increased risk of stress-related problems and suicide, an increased focus on the psychosocial work environment is warranted. The only meta-analysis on the relationship between psychosocial hazards and mental health in the construction industry (3) shows that low job support, job insecurity, and role overload were the psychosocial hazards most strongly associated with mental health problems for European construction workers. Furthermore, they found that job burnout was most strongly associated with role conflict, role ambiguity (i.e., low role clarity), interpersonal conflict, and low job support. They did not find any substantial differences between professionals and trade workers regarding adverse effects on mental health caused by psychosocial hazards.

Workplace interventions to improve the psychosocial work environment could improve working well-being, such as stress and burnout (11). Still, studies targeting the organisational level (i.e., work environment, policy) to enhance mental health within the construction industry are scant (12, 13). However, Hulls et al. (13) recently published a systematic review on workplace interventions to improve employee health and well-being in male-dominated industries. They conclude that interventions should target the organisational level rather than the individual, examine long-term effects (>12 months), and align the intervention outcomes with business activities. Other studies have also highlighted aligning the intervention with the workplace's core tasks to enhance implementation (14). Further, occupational health interventions to improve mental health in various settings (i.e., health care, school) underline the importance of the implementation process as low implementation fidelity (i.e., the extent to which the intervention was delivered according to plan) is often reported (15, 16). Reasons for low implementation fidelity could be a bad fit of the intervention to the context (17) or lack of support from the management (18). Tailoring interventions to ensure a good fit is crucial to safeguard acceptability and readiness for change, critical factors for successful implementation. Researchers co-creating their intended intervention with different stakeholders is one suggested method to meet these criteria (17, 19). Co-creating interventions mean that researchers, together with end-users and other relevant stakeholders, discuss the agenda and goals, explore the needs of end-users, decide on a format for the collaboration and design the intervention (20). Hence, co-creation will enhance participation among end-users and other stakeholders. Fox et al. (11) recently proposed a participatory process as an essential driver of well-being in occupational health interventions.

Our objective was to evaluate the effectiveness of a co-created occupational health intervention within the construction industry and evaluate the implementation fidelity. The research questions were: Was the intervention implemented as intended (i.e., fidelity)? Was there a difference in self-reported symptoms of stress between the intervention and control group over time? Was there a difference in reported role clarity, quantitative demands, team effectiveness, psychosocial safety climate, staffing, and planning between the intervention and control group over time.

## Methods

### Study design and study population

This study reports the effectiveness and implementation fidelity of a 2-year controlled trial. The trial is registered in the ISRCTN clinical trial registry (ISRCTN16548039), and the study has been approved by the Swedish Ethical Review Authority (2019-02662).

Two regions within a large Swedish construction company were recruited. Two large construction companies in Sweden were contacted, and after a few meetings, one of them agreed to participate in the study. In collaboration with representatives from the company, we did a short listing of eligible branches and regions. The building construction branch was chosen as the context for the intervention. The national health and safety manager was responsible for informing the regions about the study and looking for potential participants. One region (employees = 360) applied to participate in the study. We matched a control group (another region) from the same branch and of similar size (*N* > 300). Randomization was not viable because the intervention region wanted all groups (construction projects) to receive the intervention. Details on participant eligibility criteria, and study setting are published elsewhere (21). The gender distribution in the regions was approximately 80% men and 20% women. Professionals accounted for around two-thirds of the study population, while trade workers accounted for one-third.

### Intervention and implementation strategy

**Figure 1:**
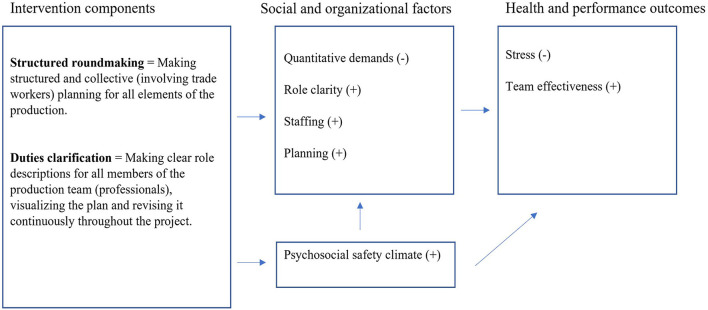
Program logic of expected order of change (21).

We co-created the program logic and implementation strategy mainly with the construction company's Health and Safety Advisory Board (HSB). HSB was an existing group comprising representatives from all organisational levels and districts, including union representatives. Details on the co-creation process are published elsewhere (21, 22). The discussions were informed by a needs assessment (i.e., interviews and a questionnaire) showing a need for improvement regarding role clarity, quantitative demands, and stress. Results also revealed that professionals were worse off than trade workers regarding almost all measured psychosocial work factors and stress. Therefore, the region chose intervention components mainly targeting professionals. The intervention components were part of their core tasks but were implemented to various degrees. Also, the construction company had never viewed the intervention components as factors to focus on to reduce stress. Hence, the representatives chose intervention components targeting performance and health in tandem. In Figure 1 we outline the program logic of the intervention describing the order in which changes are expected to occur. Also, all outcomes and the intervention components are outlined. The organisation had manuals for how to carry out the intervention components. For example, the aim of structured roundmaking is described in one of the manuals accordingly: “The aim of structured roundmaking is for the first-line manager to plan for the upcoming working procedures and remove obstacles to create trouble-free production. By continuously following up on site, the routine enhances control over the project's quality, safety and time plan for the first-line manager”.

The Production Academy (i.e., the implementation support) was implemented to increase adherence to the intervention components and included four modules with various themes on project management. We intended to enroll all construction projects (i.e., groups) in the implementation support; however, since the pandemic hindered physical meetings, the management chose to start with the four largest groups. Yet, all projects were encouraged to perform the intervention components.

The behavior change wheel, a framework to facilitate the design and description of behavior change interventions, guided the theory behind the implementation support (23). The functions of education and modeling mainly directed the content. In addition to the educational elements, the intention was for the different projects to visit each other sites and learn through observing different project management routines, such as structured roundmaking. Module one focused on production management, how to work with a weekly structure and continuous improvement strategies for managers. Module two focused on leadership and structured roundmaking. The third module dealt with leadership and how to perform daily briefings. The final module focused on leadership and how to perform time plans.

Members of the organisation mainly delivered the modules; however, an external consultant was partly responsible for the first module. The four modules were delivered accordingly: (1) A full-day face-to-face workshop plus a 2-h follow-up on Teams, (2) A full day on Teams plus a 2-h follow-up on Teams, (3) 3 h on Teams, (4) 3 h on Teams. The participating projects were encouraged to discuss their status regarding structured roundmaking and set up goals. All managers from the projects were invited. Further information about the design and theory behind the implementation strategy can be found elsewhere (21, 22).

### Data collection and outcome measures

#### Implementation fidelity

Fidelity can be measured in different ways (24). We measured adherence to the intervention (21) and the dose delivered. Hence, we assessed to what degree end-users performed structured roundmaking and duties clarification before and after the study and to what extent the implementation support was delivered. We planned to evaluate adherence with a questionnaire (21) completed by each construction project's management team and observations, but this procedure was not feasible due to the pandemic. However, the Operational manager continuously evaluated the intervention components at the construction project level using pre-set criteria. Thus, we used these ratings to assess adherence at an aggregated, regional level. For details on the pre-set criteria used for the evaluation, see Appendix A. Dose delivered of the Production Academy (i.e., implementation support) was assessed using attendance lists from the workshops.

#### Effectiveness evaluation

Primary and secondary outcomes were assessed at baseline, at 12 and 24 months, using an online survey distributed during working hours. The primary outcome of stress was measured with the Copenhagen Psychosocial Questionnaire (COPSOQ) III (25, 26). The scale has three items: (1) How often have you had problems relaxing? (2) How often have you been irritable? (3) How often have you been tense? The items are preceded by “These questions are about how you have been during the last four weeks.” The response categories for the three stress items range from (1) “all the time” to (5) “not at all”. For the analyses, we converted the scale from 1–5 to 0–100 (26). Secondary outcomes were role clarity (26), quantitative demands (26), team effectiveness (27), psychosocial safety climate (28), staffing, and planning (7). The measurement details of all secondary outcomes are shown in Appendix A.

### Statistical analysis

We measured fidelity by using descriptive statistics for the two intervention components before and after the study. Participants' outcome and demographic characteristics at baseline are presented as frequencies with percentages and mean with SD. The scales role clarity and planning were not normally distributed; thus, we transformed them using the square root function. We applied likelihood-based, mixed-effects repeated measures analyses to account for the dropout during follow-up (29). However, the analyses are valid only when the dropout pattern is missing at random (MAR). Since MAR is an assumption that is impossible to verify statistically (30, 31) and it is recommended to perform sensitivity analysis using different missing not at random (MNAR) mechanisms (32, 33), we applied pattern mixture models (PMM) (32, 34). Hence, we identified missing data patterns (MDP) to model the missing data distribution, and we created dummy variables named MDP1, MDP2, etc. Next, we applied Marginal Means Models (MMM) (i.e., Linear Mixed Model with fixed effects only) to evaluate if each missing data pattern predicted the outcome variable or interacted with time to predict changes in the outcome variable over time. The missing data patterns or the interaction between MDP and time that predicted the outcome were kept in the final model (34).

Marginal Means Models were applied to examine outcome changes from baseline to 12 and 24 months in the intervention group compared to the control group. Group and time variables were treated as fixed factors. We used the interaction of group and time as an indicator of the intervention effect at the different, discrete time points. We tested for the potential confounding variables age, gender, role seniority, job seniority, and education in two steps. First, the variables were tested univariate with the outcome. We continued the procedure only if the beta estimate for the potential confounder was statistically significant. Next, if the regression coefficient of group (intervention vs. control) or the interaction term between group and time changed by more than 20 %, the confounder was kept in the model.

Further, as the intervention component duties clarification targeted only the professionals, and structured roundmaking primarily targeted first-line managers, we performed sensitivity analyses. However, team effectiveness was not applicable, as only professionals were asked about these scale items. We aimed to examine statistically significant and clinically meaningful (i.e., noticeable differences for the individual) effects when interpreting the results (35). For COPSOQ, a change of +/- 5 is considered a noticeable difference (26, 36). *P*-values < 0.05 were considered statistically significant. We used IBM SPSS Statistics 28 to conduct the analyses for this study.

## Results

### Participants

**Figure 2:**
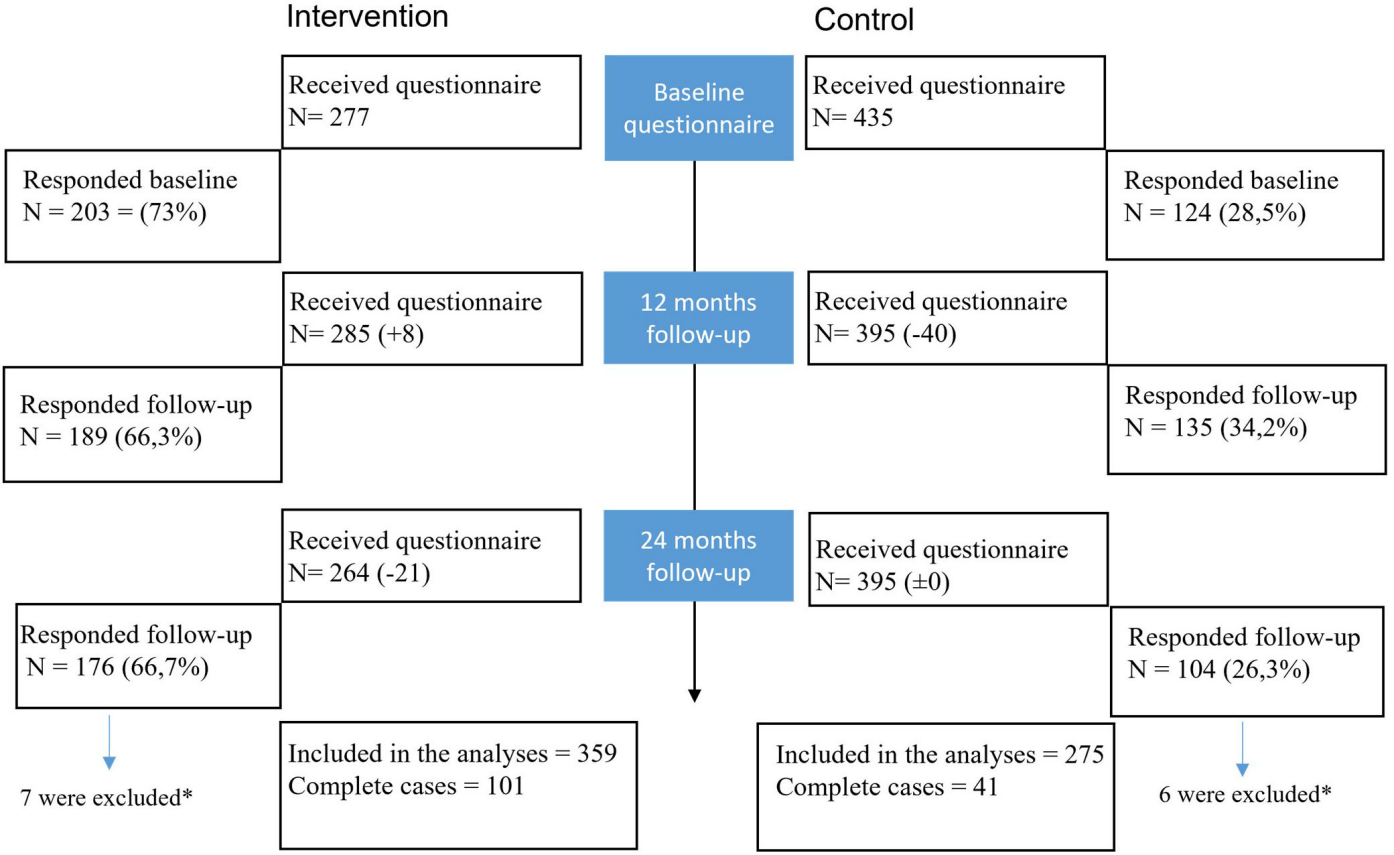
Flow chart depicting the number of workers receiving and responding to the questionnaires at the different time points. *They were excluded from the analysis as they had only responded to the last follow-up and were hired less than two years ago.

Figure 2 outlines the response rates and the number of workers included in the analysis at baseline and the two follow-ups. The response rates were higher in the intervention group, generating 101 complete cases vs. 41 in the control group. We sent the questionnaire to all employees at all time points, not restricting the sample only to employees responding at baseline. To ensure all included participants were exposed to the intervention, we excluded persons who only responded at the last follow-up and were hired < 2 years ago. We did the same for the control group to ensure the participants had been exposed to the work environment long enough to perceive it accurately. Thus, we included 359 and 275 workers in the analysis from the intervention and control groups, respectively.

Outcome and demographical characteristics of the workers at baseline are presented in Table 1. The participants' background characteristics at baseline for the intervention and control groups differed regarding age and job seniority. Participants from the control group were older and had worked longer in the organisation. The groups were equivalent regarding gender, education, role seniority, and proportion of professionals vs. trade workers and function among professionals. For the outcomes, the groups differed significantly regarding stress, quantitative demands and PSC. For all three outcomes the intervention group had worse results, i.e., higher self-reported stress, quantitative demands and lower results on PSC.

**Table 1:** Outcome and demographical characteristics at baseline of the workers in the intervention and control group.

**Variables**	**Intervention group** ***N*** = **203**				**Control group** ***N*** = **124**			
	**Mean**	**SD**	***N***	**%**	**Mean**	**SD**	***N***	**%**
**Age** (years)^*^	39, 4^*^	12, 5			44, 2	12, 9		
**Male**			162	79, 4			104	83, 9
**Education** Elementary school (9 years) Upper elementary school (> 9years) University/college Other			7 95 94 7	3, 4 46, 8 46, 3 3, 4			10 66 44 4	8, 2 54, 1 34, 4 3, 3
**Function among professionals** First line manager Site manager Project manager Project or production engineer			54 31 21 34	26, 6 15, 3 10, 3 16, 7			20 21 17 16	16, 1 16.9 13, 7 12, 9
**Trade workers**			62	30, 4			50	40,3
**Professionals**			141	69, 6			74	59,7
Job seniority^*^ < 2 2–5 >5			34^*^ 66 102	16, 8 32, 7 50, 5			14 17 91	11, 5 13, 9 74, 6
**Role seniority** < 2 2–5 >5			14 39 147	7 19, 5 73, 5			9 13 97	7, 6 10, 9 81, 5
**Stress** (0–100)^*^	34, 6^*^	18, 2			29, 3	18, 25		
**Quantitative demands** (0–100)^*^	43, 1^*^	20, 4			36, 7	17, 6		
**Role clarity** (0–100)	74, 9	16, 1			73, 7	17, 3		
**Planning** (1–3)	2, 4	0, 6			2, 4	0, 6		
**Staffing** (1–25)	14, 7	5, 6			15, 3	5, 7		
**Team effectiveness** (1–5)	3, 9	0, 6			3, 8	0, 75		
**Psychosocial safety climate** (1–5)^*^	3, 5^*^	0, 8			3, 1	0, 8		

We used independent t-tests for continuous variables and Pearson's chi-square test for categorical variables to compare the baseline characteristics between groups. SD = standard deviation.

* p < 0.05.

### Implementation fidelity

The organisation invited the four largest projects, i.e., teams, to attend the implementation support, and all groups accepted to participate. Four to six members from each team were present on all occasions. Hence, the implementation support was delivered according to plan (i.e., high fidelity) for those participating even though only four teams were enrolled. The organisation decided to postpone the delivery of the Production Academy to additional teams due to the pandemic.

Further, the results show that the adherence to both intervention components increased slightly during the trial. The assessments made by the organisation show that the mean value for Duties clarification changed from 3, 55 to 4 on a 1–5 scale. Structured roundmaking changed from 1, 75 to 1, 82 for the projects not included in the Production Academy and from 2, 6 to 4 for the enrolled projects. Appendix B outlines the details of the project ratings.

### Effects of the intervention

The results of the statistical analyses showed that there were no differences in any outcome between the groups over time, see Table 2. Therefore, we did not proceed with any analyses of confounding background variables. However, of the six MDP:s, four predicted one or several outcomes and thus were included in the final models. For detailed information about the different MDP:s and their effect on the outcomes, see Appendix C. All outcomes except role clarity deteriorated during the trial for the intervention and the control group. Yet, the control group worsened slightly more than the intervention group in all outcomes except for planning. Looking at clinically meaningful results (i.e., noticeable differences for the individual), the mean value for self-reported stress increased by five or more scale points in the intervention (+5) and the control (+6.1) group, respectively. We found a similar pattern for quantitative demands. Thus, these outcomes appear to have deteriorated noticeably in both groups.

**Table 2:** Adjusted mean values and standard deviations (SD) at all time points for the intervention and control groups.

**Variable**	**Intervention group**		**Control group**		**Model**	
	**Mean**	**SD**	**Mean**	**SD**	**Beta**	**CI**	**p**
**Stress**^**1**^ (0–100)	
Baseline	34.8	18, 5	29.5	17, 8	−1.82	−5.81–2.16	0.369
12 months	36.2	23, 4	31.5	22, 1	−7.04	−12.7–(-1.37)	0.015
24 months	39.8	22, 1	35.6	19, 7	
Intervention vs. control (group)					4, 17	−0.290–8,6	0.067
Interaction (group 0^*^time 0)					1.17	−3, 62–5.97	0.63
Interaction (group 0^*^time 1)					0.58	−4.13–5.29	0.81
**Role clarity**^**2**^ (0–100)	
Baseline	73.6	18, 5	73.5	16, 9	0.38^*^	0.052–0.719	0.023
12 months	74.1	17, 9	71.5	17, 4	0.05	−0.176–0.267	0.69
24 months	76.3	15, 6	73.9	15, 1	
Intervention vs. control (group)					0.16	−0.088–0.399	0.21
Interaction (group 0^*^time 0)					0.15	−426–0.127	0.29
Interaction (group 0^*^time 1)					0.04	−0.231–0.311	0.77
**Quantitative demands**^**3**^ (0-100)	
Baseline	40.8	21, 4	36.1	18, 9	−3.21	−7.26−0.824	.12
12 months	40.3	23, 4	37.6	18, 6	−1.88	−5.82 – 2.06	.35
24 months	46.1	18, 1	42.7	17, 6	
Intervention vs. control (group)					3.44	-.903 – 7.77	0.12
Interaction (group 0^*^time 0)					1.3	−3.58 – 6.17	0.60
Interaction (group 0^*^time 1)					−0.69	−5.52 −4.13	0.78
**Team effectiveness** (1–5)	
Baseline	3.9	0, 70	3.9	0, 67	0.09	−0.092 −0.266	0.34
12 months	4.0	0, 66	4.0	0, 63	0.18	0.006 −0.349	0.04
24 months	3.9	0, 62	3.8	0, 62	
Intervention vs. control (group)					0.09	−0.103 −0.282	0.36
Interaction (group 0^*^time 0)					-0.11	−0.330 −0.109	0.33
Interaction (group 0^*^time 1)					−0.06	−0.273−0.156	0.6
**Psychosocial safety climate** (1–5)	
Baseline	3.4	0, 85	3.2	0, 78	0.12	−0.072 −0.316	0.22
12 months	3.4	0, 82	3.3	0, 81	0.22	0.049 −0.383	0.01
24 months	3.3	0, 78	3.0	0, 78	
Intervention vs. control (group)					0.21	0.012 −0.409	0.04
Interaction (group 0^*^time 0)					0.06	−0.179 −0.307	0.60
Interaction (group 0^*^time 1)					−0.07	−0.283 −0.133	0.48
**Planning**^**1**^ (1-3)	
Baseline	2.4	0, 57	2.4	0, 56	0.01^*^	−0.047−0.057	0.85
12 months	2.5	0, 69	2.5	0, 69	0.11	0.042−0.180	0.00
24 months	2.2	0, 65	2.3	0, 59	
Intervention vs. control (group)					−0.02	−0.066−0.033	0.5
Interaction (group 0^*^time 0)					0.01	−0.055−0.071	0.80
Interaction (group 0^*^time 1)					0.00	−0.056−0.056	0.99
**Staffing** (1–25)	
Baseline	14.6	5, 39	15.2	5, 35	1.6	0.356 – 2.88	0.012
12 months	15.2	5, 50	15.5	5, 34	1.9	0.664 – 3.12	0.00
24 months	13	5, 31	13.6	5, 32	
Intervention vs. control (group)					-0.56	−1.89 -.768	0.41
Interaction (group 0^*^time 0)					0.03	−1.54 – 1.60	0.97
Interaction (group 0^*^time 1)					0.30	−1.24 – 1.85	0.70

Beta values, confidence intervals (CI), and *p*-values for the estimates of time, group, and the interaction of time by group.

1 Adjusted for MDP4,

2 djusted for MDP1,

3 Adjusted for MDP2, and 6.

* Values of a square root variable.

To explore the effects of the intervention on the occupational groups mainly involved in duties clarification and structured roundmaking, we ran a sensitivity analysis. The results show a similar trend for professionals and first-line managers as for the whole group. No significant differences were observed over time, and all outcomes except role clarity worsened for both the intervention and control groups (see Table 3). However, comparing changes in mean values over time, we identified a positive tendency for the intervention group compared to the control group regarding role clarity. The professionals in the intervention group improved by 5, 7 points (control +1, 9) and the first-line managers by 6, 2 points (control +0, 2). Hence, role clarity appears to have improved noticeably for professionals and first-line managers in the intervention group (26, 36). The improvement could not be observed in the control group. Also, there was a tendency toward an interaction of group by time regarding planning ifor first-line managers (β = -.12, CI = −0.253–0.021, *p* = 0.10), indicating a buffering effect of the intervention as the control group decreased more than the intervention group.

**Table 3:** Adjusted mean values and standard deviations (SD) for professionals and first-line managers at all time points for the intervention and control groups.

	**Professionals**							**First-line managers**						
**Variable**	**Intervention group**		**Control group**					**Intervention group**		**Control group**				
	**Mean**	**SD**	**Mean**	**SD**	**Beta**	**CI**	***p***	**Mean**	**SD**	**Mean**	**SD**	**Beta**	**CI**	***p***
**Stress**^**1**^ **(0–100)**	
Baseline	35.9	17, 9	32.2	17, 2	−0.74	−5.5 – 4.02	0.76	38.8	19, 2	29.9	18, 5	−0.44	−10.63 – 9, 75	0.93
12 months	37.3	22, 9	33.4	20, 8	−4.36	−11.2 – 2.44	0.21	38.9	24, 1	31.0	23, 2	−5.5	−18 – 7, 90	0.42
24 months	39.8	22, 0	36.0	19, 0				43.0	23, 1	33.5	19, 6	
Intervention vs. control (group)					3.86	−1.41 – 9.14	0.15					9.53	−0.996 – 20.06	0.31
Interaction (group 0^*^time 0)					−0.13	−5.77 – 5.51	0.96					−0.61	−12.24 – 11.01	0.92
Interaction (group 0^*^time 1)					0.10	−5.44 – 5.63	0.97					−1.71	−12.58 – 9.16	0.76
**Role clarity**^**2**^ **(0–100)**	
Baseline	72.7	19, 1	72.2	16, 7	0.30	−0.100 −0.693	0.14	69.9	19, 3	65.5	16, 6	−0.04^*^	−0.785−0.704	0.91
12 months	74.2	18, 4	71.9	17, 1	.06	−0.218 −0.336	0.68	72.0	19, 6	69.3	17, 7	0.28	−0.245−0.795	0.30
24 months	78.4	15, 9	74.1	15, 4				76.1	15, 9	65.7	15, 2	
Intervention vs. control (group)					0.28	−0.030 −0.594	0.08					0.67	0.101 – 1.245	0.02
Interaction (group 0^*^time 0)					−2.5	−0.590 −0.093	0.15					−0.37	−1.01−0.276	0.26
Interaction (group 0^*^time 1)					−0.10	−0.439 −0.229	0.54					−0.53	−1.13 −0.067	0.08
**Quantitative demands**^**3**^ **(0–100)**	
Baseline	45.1	20, 2	37.3	17, 3	−5.1	−9.42 –(-0.771)	0.021	43.8	22, 0	31.5	17, 3	−7.8	−16.73 – 1.08	0.08
12 months	44.9	23, 3	40.1	18, 3	−3.4	−7.59 −0.797	0.11	44.9	22, 4	41.0	17, 1	−1.3	−9.60 – 7.04	0.76
24 months	52.0	15, 9	45.4	15, 7				53.7	15, 9	41.3	15, 6	
Intervention vs. control (group)					6.6	1.73 – 11.45	0.008					12.5	3.36 – 21.54	0.01
Interaction (group 0^*^time 0)					1.2	−3.97 – 6.38	0.65					−1.16	−10.33 – 10.01	0.98
Interaction (group 0^*^time 1)					−1.7	−6.83 – 3.36	0.50					−8.5	−18.09 – 1.07	0.08
**Psychosocial safety climate**	
Baseline	3.5	0, 75	3.3	0, 72	0.13	−0.096−0.357	0.26	3, 5	0, 76	3.4	0, 74	0.37	−0.088 −0.833	0.11
12 months	3.6	0, 75	3.4	0, 72	0.22	0.27−0.416	0.03	3.7	0, 76	3.5	0, 76	0.41	0.030 −0.787	0.03
24 months	3.4	0, 73	3.2	0, 72				3.2	0, 75	3.1	0, 73	
Intervention vs. control (group)	−0.1		−0.1		0.22	−0.009 −0.443	0.06					0.18	−0.252 −0.608	0.42
Interaction (group 0^*^time 0)					0.02	−0.261 −0.296	0.90					−0.11	−0.649 −0.420	0.67
Interaction (group 0^*^time 1)					−0.01	−0.254 −0.225	0.90					0.03	−0.410 −0.473	0.89
**Planning**^**1**^ **(1–3)**	
Baseline	2.5	0, 55	2.6	0, 54	0.03	−0.030 −0.091	.33	2.4	0, 56	2.5	0, 55	0.13^*^	0.009 −0.249	0.04
12 months	2.6	0, 69	2.6	0, 63	0.10	0.015 −0.181	.02	2.6	0, 68	2.5	0, 66	0.14	−0.005 −0.286	0.06
24 months	2.4	0, 67	2.4	0, 59				2.3	0, 66	2.1	0, 58	
Intervention vs. control (group)					0.01	−0.046 −0.066	0.72					0.08	−0.022 −0.191	0.12
Interaction (group 0^*^time 0)					−0.04	−0.112 −0.033	0.29					−0.12	−0.253 −0.021	0.10
Interaction (group 0^*^time 1)					−0.02	−0.083 −0.049	0.62					−0.04	−0.162 −0.072	0.45
**Staffing (1–25)**	
Baseline	15.4	5, 22	16.7	5, 16	2.3	0.782 – 3.93	0.003	14.0	5, 37	16.2	5, 33	4.5	1.26 – 7.69	0.01
12 months	16.2	5, 20	16.9	5, 19	2.5	1.02 – 4.07	0.001	15.7	5, 38	16.5	5, 42	4.7	1.73 – 7.68	0.00
24 months	13.8	5, 16	14.3	5, 12				12.3	5, 33	11.7	5, 25	
Intervention vs. control (group)					−0.50	−2.10 −0.101	0.54					0.60	−2.49– 3.68	0.70
Interaction (group 0^*^time 0)					−0.79	−2.71–1.14	0.42					−2.84	−6.36 −0.884	0.13
Interaction (group 0^*^time 1)					−0.17	−2.07 – 1.73	0.86					−1.37	−4.86–2.13	0.44

^1^Adjusted for MDP4, ^2^Adjusted for MDP1, ^3^Adjusted for MDP2 and 6, ^1^Adjsuted for MDP4.

* Values of a square root variable. Beta values, confidence intervals (CI), and p-values for the estimates of time, group, and the interaction of time by group.

## Discussion

This study aimed to evaluate the effectiveness of a co-created intervention on psychosocial work factors and self-reported symptoms of stress within the construction industry and evaluate the implementation fidelity. The results show that the implementation fidelity (i.e., intervention adherence and dose delivered) was fairly high. However, we found no significant differences between the intervention and control group for any outcomes over time. Our results show that all outcomes, excluding role clarity, deteriorated for both the intervention and the control group. Yet, we observe a noticeable improvement in role clarity for professionals, especially first-line managers, the groups mainly targeted by the intervention.

Our results align with the program logic for this project, which predicted that the implementation of structured roundmaking and duties clarification would improve role clarity. However, the theory of health, indicating that high role clarity would be associated with low stress (37), was contradicted. Instead, stress seemed highly related to quantitative demands, which markedly increased in all groups irrespective of occupation.

How can we understand the noticeable increase in stress and quantitative demands during the trial? The pandemic would be one obvious explanation as the study baseline was in December 2019, and the last follow-up was in December 2021. However, there is a lack of research to confirm or disprove this trend. Nevertheless, the Swedish work environment authority (38) reports increased work-related disorders between 2018–2020, mainly due to high workload. The increase was valid regardless of gender, age or occupation. Also, the construction company's employee surveys and health assessments confirm an adverse trend in the psychosocial work environment and stress for professionals and trade workers between 2020–2021. Accordingly, the unfavorable development of most outcomes in the intervention and control groups appears to be related to factors outside the organisation. Additionally, the enrolled region has not indicated that the intervention was burdensome or demanding, which has been seen in similar studies reporting adverse health effects (39, 40). Instead, the participants of the co-creation process perceived the intervention components and the implementation strategy as relevant and well-tailored to the organisation's context (22). Thus, given the positive perceptions and as both the intervention and the control group deteriorated, we do not believe the adverse effect was due to the intervention. Instead, the intervention appears to have had some buffering effect.

Despite the negative development in many outcomes, role clarity increased noticeably for the professionals and the first-line managers in the intervention group. Hence, there are indicators that the intervention components duties clarification and structured roundmaking positively affected this outcome for these groups. Yet, the increase in role clarity did not have the expected positive effect on symptoms of stress. One explanation for this could be the time aspect. The main growth in role clarity took place during the last year (i.e., 2020–2021), and a possible effect of that increase on stress might emerge later. Organisational and behavioral changes take time (41), and research suggests a follow-up time of two to three years (19). As low role clarity is a predictor of stress (37) and specifically burnout among construction workers (3), our results are promising for future research to improve the construction industry's psychosocial work environment. Still, more studies with larger sample sizes need to test the intervention components' effectiveness on stress among professionals.

Low implementation fidelity is raised as one of the biggest concerns in health-promoting research in the workplace (15, 42). Not adapting the intervention to the organisation's specific conditions and the needs of end-users hindering necessary organisational and behavioral changes are suggested reasons (43). Our results show a reasonably high implementation fidelity, i.e., we could observe a behavior change for structured roundmaking and duties clarification. The aim of co-creating the intervention and the implementation plan was to ensure the intervention targeted a problem raised by the end-users (i.e., that it was relevant and meaningful) and was feasible to implement. It is reasonable to assume that the involvement of different stakeholders enabled a buy-in from both the management and end-users, facilitating readiness for change. Hence, the results of our study support the notion that co-creating occupational health interventions could be one solution for improved implementation of occupational health interventions.

### Limitations and strengths

We acknowledge the limitation of the low response rates, especially in the control group, possibly introducing attrition bias. However, we applied pattern mixture models (44) to allow the slope to differ within subgroups of different dropout patterns to account for this dropout.

Applying linear mixed models (LMM) to handle missingness is only correct if the MAR assumption is correctly understood. However, researchers commonly misunderstand the MAR assumption, rendering biased results (45). Our results showed that several of the MDP:s significantly impacted various outcomes, which justifies the use of PMM in this study.

Yet, a limitation of PMM is the decrease of power as the analysis includes additional predictors in the model (34). Also, the co-creation method contributed to power issues because the organisation chose to target mainly the professionals with the intervention. We assumed that all occupations would be involved in the intervention when we did the power calculation (21). In this research design, these types of possible misunderstandings resulting from the co-creation process are essential to consider since they may otherwise result in decreased internal validity.

Nevertheless, using co-creation is a strength of this study as the method appears to have facilitated adherence to the intervention components and thus a necessary behavioral change. Hence, co-creating the intervention and the implementation strategy seems to enable acceptability and feasibility, which are essential factors for successful implementation. Another strength of the present study is the fact that we measured fidelity and how we measured it. A recent narrative review on fidelity in workplace mental health intervention research shows that only 20% of the included studies used the word “fidelity” or a similar concept. The adherence measurement in the present study made it possible to link the changes in the outcomes to an actual intervention-related behavior change. Research suggests that adherence measurements should rely on observations rather than self-reporting (37), aligning with our planned strategy. However, because of the pandemic, we could not enter the worksites. Instead, one organisation member made the assessments in discussion with the project management team using predetermined criteria.

More, this study's external validity is a methodological consideration because we could not randomize the intervention and control group, likely rendering selection bias. The management team of the enrolled region had a high engagement in work environment improvements and a high organisational capability (46). However, we believe that the evaluated intervention components generally apply in the construction industry and that the associated improvements in role clarity are generalisable to these settings. Finally, other strengths of the design of this study are the long follow-up time, 24 months, the use of a logic model, and the fact that the management approved including the co-created intervention components in the business case.

### Concluding remarks

This is the first study evaluating the effects of structured roundmaking and duties clarification on psychosocial working conditions and mental health (stress). Our results show no significant differences between the intervention and control group over time for the primary outcome stress. However, we present evidence for the beneficial effect of duties clarification and structured roundmaking on professionals' psychosocial working conditions (role clarity). Hence, this study adds to the literature by displaying how an occupational health intervention can improve role clarity, a significant predictor of health for construction workers.

Further, the use of co-creation appears to have positively impacted implementation fidelity, i.e., adherence to the intervention components. Our results are significant as the need to identify efficient strategies for enhancing the implementation of occupational health interventions is stressed. Also, the study provides a novel methodological approach to handling high dropout rates, an increasing problem in intervention studies.

More studies are needed to evaluate these intervention components with higher power and two to three-year follow-ups. Also, we recommend researchers of future intervention studies consider the use of MNAR sensitivity analysis, of which PMM is one example. Finally, the possible increased risk for professionals and first-line managers within the construction industry to suffer from stress-related problems also needs further investigation.

## Data availability statement

The datasets presented in this article are not readily available because the datasets generated and analyzed during the current study are not publicly available due to legal restrictions. Requests to access the datasets should be directed to emma.cedstrand@ki.se.

## Ethics statement

The studies involving human participants were reviewed and approved by Swedish Ethical Review Authority (2019-02662). The patients/participants provided their written informed consent to participate in this study.

## Author contributions

EC, AN, HA, and TB designed the study. EC acquired the data, drafted the paper. EC, GJ, MA, and NS analyzed the data. All authors contributed to interpreting the results, and revised and edited the final manuscript.

## Funding

This project is funded by AFA-insurance (Reg. No. 170129).

## Conflict of interest

The authors declare that the research was conducted in the absence of any commercial or financial relationships that could be construed as a potential conflict of interest.

## Publisher's note

All claims expressed in this article are solely those of the authors and do not necessarily represent those of their affiliated organizations, or those of the publisher, the editors and the reviewers. Any product that may be evaluated in this article, or claim that may be made by its manufacturer, is not guaranteed or endorsed by the publisher.

## References

[1] Arbetsmiljöverket [Swedish Work Environment Authority]. Arbetsskador 2015 [Occupational accidents and work-related diseases 2015]. Stockholm: Swedish Work Environment Authority. (2015).

[2] Försäkringskassan [Swedish Social Insurance Agency]. Sjukfrånvaro i psykiska diagnoser: en registerstudie av Sveriges arbetande befolkning i åldern 20–69 år. Socialförsäkringsrapport 2020:8 [Sick leave due to mental disorders: a register based study of the working population 20-69 years in Sweden. Social Insurance Report 2020:8]. Stockholm: Försäkringskassan [Swedish Social Insurance Agency] (2020).

[3] Sun C Hon CKH Way KA Jimmieson NL Xia B. The relationship between psychosocial hazards and mental health in the construction industry: a meta-analysis. Saf Sci. (2022) 145:105485. 10.1016/j.ssci.2021.105485

[4] Windsor-Shellard B. Suicide by occupation, England: 2011 to 2015. Analysis of deaths from suicide in different occupational groups for people aged 20 to 64 years, based on deaths registered in England between 2011 and 2015. Office for National Statistics (2017).30929655

[5] Suicide Mortality Review Committee. Ngā Rāhui Hau Kura: Suicide Mortality Review Committee Feasibility Study 2014–15. Report to the Ministry of Health, 31 May 2016. Wellington: Suicide Mortality Review Committee (2016).

[6] Matilla-Santander N Blazevska B Carli V Hadlaczky G Linnersjö A Bodin T. The relation between occupation, gender dominance in the occupation and workplace and suicide in Sweden: a longitudinal study. BMJ Open. (2022) 12:e060096. 10.1136/bmjopen-2021-060096 35738642 PMC9226951

[7] Stenberg M. Bortom noll: En hälsofrämjande byggbransch [Beyond zero: A health promoting construction industry]. Luleå: Luleå tekniska universitet [Luleå University of Technology]. (2016).

[8] Leung M-Y Liang Q Olomolaiye P. Impact of job stressors and stress on the safety behavior and accidents of construction workers. J Manag Eng. (2016) 32:04015019. 10.1061/(ASCE)ME.1943-5479.0000373

[9] Clarke S. An integrative model of safety climate: Linking psychological climate and work attitudes to individual safety outcomes using meta-analysis. J Occup Organ Psychol. (2010) 83:553–78. 10.1348/096317909X452122

[10] Anwer S Li H Antwi-Afari MF Wong AYL. Associations between physical or psychosocial risk factors and work-related musculoskeletal disorders in construction workers based on literature in the last 20 years: a systematic review. Int J Ind Ergon. (2021) 83:103113. 10.1016/j.ergon.2021.103113

[11] Fox KE Johnson ST Berkman LF Sianoja M Soh Y Kubzansky LD. Organisational- and group-level workplace interventions and their effect on multiple domains of worker well-being: a systematic review. Work Stress. (2022) 36:30–59. 10.1080/02678373.2021.1969476

[12] Lee NK Roche A Duraisingam VA. Fischer J, Cameron J. Effective interventions for mental health in male-dominated workplaces. Ment Health Rev J. (2014) 19:237–50. 10.1108/MHRJ-09-2014-0034

[13] Hulls PM Richmond RC Martin RM Chavez-Ugalde Y de Vocht F. Workplace interventions that aim to improve employee health and well-being in male-dominated industries: a systematic review. Occup Environ Med. (2021). 10.1186/s13643-019-1260-9 34035181 PMC8785069

[14] Framke E Sørensen OH Pedersen J Rugulies R. Effect of a participatory organizational-level occupational health intervention on short-term sickness absence: a cluster randomized controlled trial. Scand J Work Environ Health. (2016) 42:192–200. 10.5271/sjweh.3559 27046654

[15] Wolfenden L Yoong SL. Workplace wellness programmes to improve health. Lancet Public Health. (2021) 6:e625–e. 10.1016/S2468-2667(21)00184-5 34454641

[16] Montano D Hoven H Siegrist J. Effects of organisational-level interventions at work on employees' health: a systematic review. BMC Public Health. (2014) 14:135. 10.1186/1471-2458-14-135 24507447 PMC3929163

[17] von Thiele Schwarz U Nielsen K Edwards K Hasson H Ipsen C Savage C. How to design, implement and evaluate organizational interventions for maximum impact: the Sigtuna Principles. Eur J Work Org Psychol. (2021) 30:415–27. 10.1080/1359432X.2020.1803960 34518756 PMC8432268

[18] Lundmark R Hasson H von Thiele Schwarz U Hasson D Tafvelin S. Leading for change: line managers' influence on the outcomes of an occupational health intervention. Work Stress. (2017) 31:276–96. 10.1080/02678373.2017.1308446

[19] Moore GF Evans RE Hawkins J Littlecott H Melendez-Torres GJ Bonell C. From complex social interventions to interventions in complex social systems: future directions and unresolved questions for intervention development and evaluation. Evaluation. (2019) 25:23–45. 10.1177/1356389018803219 30705608 PMC6330692

[20] Skelton DA Altenburg TM. Cardon G Chinapaw MJM. Framework, principles and recommendations for utilising participatory methodologies in the co-creation and evaluation of public health interventions. Res Involv Engagem. (2019) 5:2. 10.1186/s40900-018-0136-9 30652027 PMC6327557

[21] Cedstrand E Nyberg A Bodin T Augustsson H Johansson G. Study protocol of a co-created primary organizational-level intervention with the aim to improve organizational and social working conditions and decrease stress within the construction industry - a controlled trial. BMC Public Health. (2020) 20:424. 10.1186/s12889-020-08542-7 32228509 PMC7106574

[22] Cedstrand E Molsted Alvesson H Augustsson H Bodin T Bodin E Nyberg A. Co-creating an occupational health intervention within the construction industry in sweden: stakeholder perceptions of the process and output. Int J Environ Res Public Health. (2021) 18:12872. 10.3390/ijerph182412872 34948487 PMC8700815

[23] Michie S van Stralen MM West R. The behaviour change wheel: a new method for characterising and designing behaviour change interventions. Implement Sci. (2011) 6:42. 10.1186/1748-5908-6-42 21513547 PMC3096582

[24] Dusenbury L Brannigan R Falco M Hansen WBA. review of research on fidelity of implementation: implications for drug abuse prevention in school settings. Health Educ Res. (2003) 18:237–56. 10.1093/her/18.2.237 12729182

[25] Burr H Berthelsen H Moncada S Nübling M Dupret E Demiral Y. The third version of the copenhagen psychosocial questionnaire. Saf Health Work. (2019) 10:482–503. 10.1016/j.shaw.2019.10.002 31890332 PMC6933167

[26] Berthelsen H Westerlund H Bergstrom G Burr H. Validation of the copenhagen psychosocial questionnaire version III and establishment of benchmarks for psychosocial risk management in Sweden. Int J Environ Res Public Health. (2020) 17:3179. 10.3390/ijerph17093179 32370228 PMC7246423

[27] Maynard MT Mathieu JE Rapp TL Gilson LL. Something(s) old and something(s) new: modeling drivers of global virtual team effectiveness. J Organ Behav. (2012) 33:342–65. 10.1002/job.1772

[28] Berthelsen H Ertel M Geisler M Muhonen T. Validating the psychosocial safety climate questionnaire – integration of findings from cognitive interviews in Germany and Sweden. Scand J Work Organ Psychol. (2019) 4:9. 10.16993/sjwop.85

[29] Mallinckrodt CH Clark WS David SR. Accounting for dropout bias using mixed-effects models. J Biopharm Stat. (2001) 11:9–21. 10.1081/BIP-100104194 11459446

[30] Rhoads C. Problems with tests of the missingness mechanism in quantitative policy studies. Statistics Politics Policy. (2012) 3. 10.1515/2151-7509.1012

[31] Molenberghs G Beunckens C Sotto C Kenward MG. Every missingness not at random model has a missingness at random counterpart with equal fit. J Roy Stat Soc Ser B (Stat Method). (2008) 70:371–88. 10.1111/j.1467-9868.2007.00640.x

[32] Little RJA. Modeling the drop-out mechanism in repeated-measures studies. J Am Stat Assoc. (1995) 90:1112–21. 10.1080/01621459.1995.10476615

[33] Schafer JL Graham JW. Missing data: our view of the state of the art. Psychol Methods. (2002) 7:147–77. 10.1037/1082-989X.7.2.147 12090408

[34] Son H Friedmann E Thomas SA. Application of pattern mixture models to address missing data in longitudinal data analysis using SPSS. Nurs Res. (2012) 61:195–203. 10.1097/NNR.0b013e3182541d8c 22551994

[35] Amrhein V Greenland S McShane B. Scientists rise up against statistical significance. Nature. (2019) 567:305–7. 10.1038/d41586-019-00857-9 30894741

[36] Pejtersen JH Bjorner JB Hasle P. Determining minimally important score differences in scales of the copenhagen psychosocial questionnaire. Scand J Public Health. (2010) 38 (Suppl. 3):33–41. 10.1177/1403494809347024 21172769

[37] Harvey SB Modini M Joyce S Milligan-Saville JS Tan L Mykletun A. Can work make you mentally ill? A systematic meta-review of work-related risk factors for common mental health problems. Occup Environ Med. (2017) 74:301–10. 10.1136/oemed-2016-104015 28108676

[38] Arbetsmiljöverket [Swedish Work Environment Authority]. Arbetsorsakade besvär 2020 [Work-related Disorders 2020]. Stockholm: Swedish Work Environment Authority (2021).

[39] Gupta N Wahlin-Jacobsen CD Abildgaard JS Henriksen LN Nielsen K Holtermann A. Effectiveness of a participatory physical and psychosocial intervention to balance the demands and resources of industrial workers: a cluster-randomized controlled trial. Scand J Work Environ Health. (2018) 44:58–68. 10.5271/sjweh.3689 29095478

[40] Cedstrand E Nyberg A Sanchez-Bengtsson S Alderling M Augustsson H Bodin T. A participatory intervention to improve the psychosocial work environment and mental health in human service organisations. A mixed methods evaluation study. Int J Environ Res Public Health. (2021) 18:3546. 10.3390/ijerph18073546 33805501 PMC8037176

[41] Patton GC Bond L Carlin JB Thomas L Butler H Glover S. Promoting social inclusion in schools: a group-randomized trial of effects on student health risk behavior and well-being. Am J Public Health. (1971) 96:1582–7. 10.2105/AJPH.2004.047399 16873760 PMC1551970

[42] Robroek SJW Coenen P Oude Hengel KM. Decades of workplace health promotion research: marginal gains or a bright future ahead? Scand J Work Environ Health. (2021) 47:561–4. 10.5271/sjweh.3995 34655223 PMC9058620

[43] Moore GF Evans RE. What theory, for whom and in which context? Reflections on the application of theory in the development and evaluation of complex population health interventions. SSM Popul Health. (2017) 3:132–5. 10.1016/j.ssmph.2016.12.005 29302610 PMC5742639

[44] Hedeker D Gibbons RD. Application of random-effects pattern-mixture models for missing data in longitudinal studies. Psychol Methods. (1997) 2:64–78. 10.1037/1082-989X.2.1.6430146938

[45] Magnusson K. Methodological Issues in Psychological Treatment Research: Applications to Gambling Research and Therapist Effects. [Dissertation] [Stockholm]: Karolinska Institutet. (2019).

[46] Mellor N Mackay C Packham C Jones R Palferman D Webster S. “Management standards” and work-related stress in Great Britain: progress on their implementation. Saf Sci. (2011) 49:1040–6. 10.1016/j.ssci.2011.01.010

