# Supplementary material for: Effects of a co-created occupational health intervention on stress and psychosocial working conditions within the construction industry: A controlled trial
Source: Front Public Health. 2022 Sep 23;10:973890. doi: 10.3389/fpubh.2022.973890 (PMC9542354; doi:10.3389/fpubh.2022.973890)
Supplement: Supplementary file 1 [file Data_Sheet_1.docx]

Appendix A. Description of the measurement of the secondary outcomes and background variables and the pre-set criteria for measuring adherence to the two intervention components.

# Secondary outcomes

## Quantitative demands

This dimension comes from COPSOQ III [25, 26] and includes three items: (1) Do you get behind with your work? (2) Is your workload unevenly distributed so it piles up? (3) How often do you not have time to complete all your work tasks? The response categories range from 1= "always" to 5= "never/hardly ever." For the analyses, we converted the scale from 1 – 5 to 0 – 100 [25].

## Role clarity

This dimension comes from COPSOQ III [25, 26] and includes three items: (1) Does your work have clear objectives? (2) Do you know exactly which areas are your responsibility? (3) Do you know exactly what is expected of you at work? The response categories range from (1) "to a very large extent" to (5) "to a very small extent." For the analyses, we converted the scale from 1 – 5 to 0 – 100 [25].

Psychosocial safety climate

The psychosocial safety climate scale is validated in Swedish [28] and includes four items: (1) Senior management considers employee psychological health as important as productivity, (2) Senior management show support for stress prevention through involvement and commitment, (3) In my organization, the prevention of stress involves all levels of the organization, (4) There is good communication here about psychological safety issues which affect me. The response categories range from (1) strongly disagree to (5) strongly agree.

# Additional outcomes

Team effectiveness

Team effectiveness was assessed with a scale constructed by Maynard [27]. The scale includes four items preceded with the text "For each item, please indicate your opinion regarding how effective your team was": (1) How effective is your team in using the skills of the different team members? (2) How effective was your team in generating ideas for the project? (3) How effective was your team at coordinating? (4) How effective was your team in developing its final project? The response categories ranged from (1) "not good at all" to (5) "very good." This scale has not been validated in Swedish.

Planning

Planning was assessed with one item, "Do you experience the work at your workplace as well-planned?" The response categories ranged from (1) "no, not at all" to (3) "yes, fully." Stenberg [7] used this item in a Swedish report investigating the relationship between serious workplace accidents and the work environment.

Staffing

Staffing was assessed with two self-constructed items (1) is the staffing at your workplace sufficient for the number of individuals? (2) is the staffing at your workplace sufficient regarding competence? The response categories ranged from (1) "to a very low extent" to (5) "to a great extent". For the analyses, we converted the scale from 1 – 5 to 1 – 25 by multiplying the responses from the items.

# Background variables

For **age**, we asked "How old are you (in years)" in the survey and collected data from the company registers. To assess **gender,** we included the statement "Gender identity, ie., the gender you feel like" with the three response categories: male, female or other. To assess **education** we included the statement "Highest completed education" followed by four response categories: Elementary school (9 years), Upper elementary school (> 9years), University/college or Other. **Job seniority** was assessed with the item "How long have you worked at the company," For **role seniority**, we used "How long have you worked in the occupation? The job and role seniority response categories were "<2 years", "2-5 years", ">5 years".

# Adherence to the intervention components

For Duties clarification, the pre-set criteria range from 1 – 5, where 1 means "the project lacks competence and is understaffed," and 4 means "We update the duties clarification of the work tasks in the project regularly." 5 is defined as world leading.

For structured roundmaking, 1 means "some of the first-line managers carry out structured roundmaking using a structured roundmaking list." 4 means "first-line managers use a structured roundmaking list to track which activities must be prepared and evaluated, and all first-line managers carry out structured roundmaking using a structured roundmaking list." 5 is defined as world leading.

Appendix B. Ratings of adherence to the intervention components.

Table A1. Number of projects, means and standard deviations for the ratings of the two intervention components.

|  | Before | | | |  | | | After | | | | |
| --- | --- | --- | --- | --- | --- | --- | --- | --- | --- | --- | --- | --- |
|  | N^projects^ | Mean | SD |  | |  |  | | N^projects^ | Mean | SD |  |
| **Duties clarification*** | 11 | 3,55 | 0,66 |  | |  |  | | 16 | 4 | 0.30 |  |
|  |  |  |  |  | |  |  | |  |  |  |  |
| **Structured roundmaking** |  |  |  |  | |  |  | |  |  |  |  |
| Projects not involved in the Production Academy | 4 | 1.75 | 0,83 |  | |  |  | | 8 | 1.8 | 0,82 |  |
|  |  |  |  |  | |  |  | |  |  |  |  |
|  | Before PA^2^ After PA^3^ Q4 2021 | | | | | | | | | | | |
| **Structured roundmaking** | N^projects^ | Mean | SD | N^projects^ | | Mean | SD | | N^projects^ | Mean | SD | |
| Projects enrolled in the PA | 4 | 2,6 | 0,96 | 3 | | 3.3 | 1,0 | | 2 | 4 | 1.4 |  |

PA=Production Academy, ^2^Measures taken in October 2020, ^3^Measures taken in April 2021,*One project was excluded after discussions with the organization as it was considered an outlier.

Before measures were taken during 2019 and the first quarter of 2020, no activities were implemented. After measures were taken from the second quarter of 2020 until 2022. We excluded new projects with baseline measures taken during the fourth quarter of 2021.

Appendix C. Description of the missing data patterns and their effect on the outcomes.

Pattern 1 = missing at 2 year follow up, N=67 = MDP1

Pattern 2 = missing at 1 and 2 year follow up, N= 78 = MDP2

Pattern 3 = missing at 1 year follow up, N=41 = MDP3

Pattern 4 = missing at baseline, N=51 = MDP4

Pattern 5 = missing at baseline and first follow-up, N= 45 = MDP5

Pattern 6 = missing at baseline and second follow up, N= 64 = MDP6

Table A2. P-values for the MDP:s and their interaction with time for all outcomes.

| **Outcome** | **Statistically significant patterns** | **Details significant patterns** |
| --- | --- | --- |
| Stress | Pattern 4^1^ | ^1^MDP4 = 004, TIME2 = .008 and MDP4*TIME2 = .037 |
| Sqrt_role clarity | Pattern 1^1^ | ^1^MDP1 = <.001 samt TIME0 = .028 and MDP1*TIME0 = .028 |
| Quantitaive demands | Pattern 2^1^, Pattern 6^2^ | ^1^MDP2 = .006, Time .04  ^2^MDP6=,013, Time0=,009 |
| Sqrt_planning | Pattern4^1^ | ^1^MDP4=,004 och TIME1 <,001 och MDP4*TIME1=,034 |
| Staffing | No significant patterns |  |
| Psychosocial safety climate | No significant patterns |  |
| Team effectiveness* | No significant patterns |  |

*Team effectiveness had a different pattern of missing as only professionals responded to the related items.
